# A Promising Strategy for Solvent-Regulated Selective Hydrogenation of 5-Hydroxymethylfurfural over Porous Carbon-Supported Ni-ZnO Nanoparticles

**DOI:** 10.1007/s40820-025-01847-5

**Published:** 2025-07-18

**Authors:** Rulu Huang, Chao Liu, Kaili Zhang, Jianchun Jiang, Ziqi Tian, Yongming Chai, Kui Wang

**Affiliations:** 1 National Key Laboratory for Development and Utilization of Forest Food Resources, Biomass Energy and Material Key Laboratory of Jiangsu Province, Institute of Chemical Industry of Forest Products, Chinese Academy of Forestry, Nanjing, 210042 People’s Republic of China; 2 Co-Innovation Center of Efficient Processing and Utilization of Forest Resources, Nanjing Forestry University, Nanjing, 210037 People’s Republic of China; 3 School of Materials Science and Engineering, Central South University of Forestry and Technology, Changsha, 410004 People’s Republic of China; 4 Ningbo Institute of Materials Technology and Engineering, Chinese Academy of Sciences, Ningbo, 315201 People’s Republic of China; 5 State Key Laboratory of Heavy Oil Processing, College of Chemistry and Chemical Engineering, China University of Petroleum (East China), Qingdao, 266580 People’s Republic of China

**Keywords:** Porous carbon-supported Ni-ZnO nanoparticles catalyst, Selective hydrogenation, 5-Hydroxymethylfurfural, Solvent, Proton-donating ability

## Abstract

Developing biomass platform compounds into high value-added chemicals is a key step in renewable resource utilization. Herein, we report porous carbon-supported Ni-ZnO nanoparticles catalyst (Ni-ZnO/AC) synthesized via low-temperature coprecipitation, exhibiting excellent performance for the selective hydrogenation of 5-hydroxymethylfurfural (HMF). A linear correlation is first observed between solvent polarity (E_T_(30)) and product selectivity within both polar aprotic and protic solvent classes, suggesting that solvent properties play a vital role in directing reaction pathways. Among these, 1,4-dioxane (aprotic) favors the formation of 2,5-bis(hydroxymethyl)furan (BHMF) with 97.5% selectivity, while isopropanol (iPrOH, protic) promotes 2,5-dimethylfuran production with up to 99.5% selectivity. Mechanistic investigations further reveal that beyond polarity, proton-donating ability is critical in facilitating hydrodeoxygenation. iPrOH enables a hydrogen shuttle mechanism where protons assist in hydroxyl group removal, lowering the activation barrier. In contrast, 1,4-dioxane, lacking hydrogen bond donors, stabilizes BHMF and hinders further conversion. Density functional theory calculations confirm a lower activation energy in iPrOH (0.60 eV) compared to 1,4-dioxane (1.07 eV). This work offers mechanistic insights and a practical strategy for solvent-mediated control of product selectivity in biomass hydrogenation, highlighting the decisive role of solvent-catalyst-substrate interactions.

**Graphical Abstract:**

**Supplementary Information:**

The online version contains supplementary material available at 10.1007/s40820-025-01847-5.

## Highlights

- A porous carbon-supported Ni-ZnO nanoparticles catalyst (Ni-ZnO/AC) was synthesized by low-temperature coprecipitation, demonstrating exceptional catalytic activity and stability.
- Selective hydrogenation of 5-hydroxymethylfurfural (HMF) to 2,5-bis(hydroxymethyl)furan (97.5%) or 2,5-dimethylfuran (99.5%) is achieved over Ni-ZnO/AC catalyst by solvent-tuning.
- Solvent-catalyst interaction jointly regulates hydrodeoxygenation behavior in HMF hydrogenation by modulating rate and pathway via a hydrogen shuttle mechanism.

**Supplementary Information:**

The online version contains supplementary material available at 10.1007/s40820-025-01847-5.

## Graphical abstract

**Figure:**
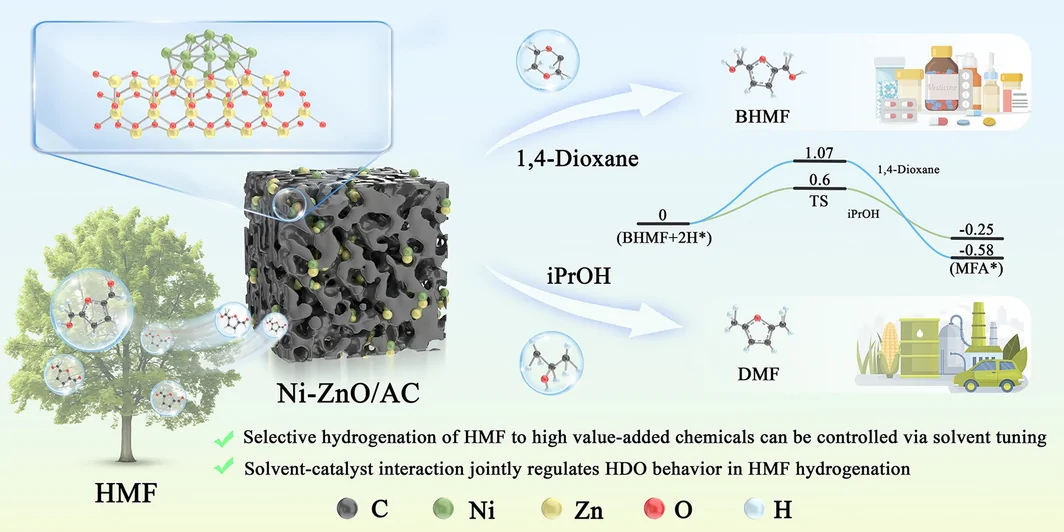
A porous carbon-supported Ni-ZnO nanoparticles catalyst (Ni-ZnO/AC) enables solvent-regulated selective hydrogenation of HMF to high-value chemicals (BHMF/DMF), among solvent-catalyst interaction modulates HDO behavior, with lower activation energy in iPrOH versus 1,4-dioxane.

## Introduction

Numerous agricultural and forestry residues can be converted into renewable fuels and high value-added chemicals through biorefining processes, offering a reliable pathway for sustainable energy development [1–3]. In biorefining, biomass platform compounds serve as intermediates for producing various high value-added chemicals. Notably, 5-hydroxymethylfurfural (HMF) rank among the “Top 10 + 4” biomass platform compounds identified by the US Department of Energy due to a critical link between biomass resources and petroleum-based industries [4, 5]. HMF possesses rich functional groups (aldehyde, hydroxymethyl and furan ring), enabling its conversion into a variety of high value-added chemicals through catalytic hydrogenation. For instance, HMF can be converted into 2,5-bis(hydroxymethyl)furan (BHMF), which is utilized as a pharmaceutical intermediate and in synthetic polyester production; 2,5-bis(hydroxymethyl)tetrahydrofuran (BHMTHF), a precursor for biopolymer monomers; 2,5-dimethylfuran (DMF), a second-generation liquid biofuel; and 1,6-hexanediol (1,6-HDO), an important precursor of bioplastics [6–10]. HMF hydrogenation involves a complex reaction network, necessitating selective activation of its functional groups to achieve the synthesis of the target products [11]. However, addressing selective hydrogenation of HMF into two or even multiple target products within a single catalytic reaction system remains an urgent challenge that must be solved.

Currently, much of the research on the catalytic hydrogenation of HMF emphasizes the precise design of catalysts with specific active sites to facilitate the efficient conversion of substrates. For instance, He et al. discovered that the Pt/OMS-2 catalyst, derived from screening Pt nanoparticles supported on several metal oxides, exhibits selectivity for the C = O bond and efficiently catalyzes the conversion of HMF to BHMF [12]. Kang et al. innovatively developed bimetallic active sites on a CeO_2_ support, creating a synergistic catalytic system comprising trace Pd atoms and plate-shaped Cu^+^ clusters with one-atom layers. In this system, the Pd atoms enable the heterogeneous dissociation activation of hydrogen, while the plate-shaped Cu^+^ clusters serve as active sites for hydrogenation, significantly enhancing the catalytic efficiency of HMF [13]. Furthermore, Wu et al. prepared a bifunctional catalyst (Br-Pd/Al_2_O_3_) through bromobenzene pretreatment, achieving stable catalytic hydrogenation of HMF by synergistically incorporating metal sites and Brønsted acid sites on Al_2_O_3_ [14]. Notably, despite these significant breakthroughs in catalyst structure design, there remain critical gaps in the existing research on the liquid-phase hydrogenation of HMF, particularly regarding the exploration of key mechanisms such as solvent effect and the regulation of reaction pathways during the catalytic process. Solvents play several important functions in hydrogenation. First, they promote the mixing of reaction substrates and enhance their contact with the catalyst, thereby increasing the likelihood of collisions and subsequent reactions [15, 16]. Second, solvents can enable the reaction to proceed at lower temperatures and pressures, effectively reducing the activation energy required for the reaction [17, 18]. Furthermore, solvents may interact with the active sites on the catalyst, stabilize surface intermediates, or facilitate proton transfer from external hydrogen donors. The selection and comparison of solvents represent another viable approach to improve the efficiency of selective hydrogenation [19–21]. Previous studies have shown that solvent properties, particularly polarity and protic/aprotic character, can significantly influence catalytic hydrogenation reactions by affecting the strength of Lewis acid sites, stabilizing reactive intermediates, and altering the reaction rate [22]. For example, in the hydrodechlorination of chlorinated organics over Pt/C catalysts, notable differences in both conversion rate and intermediate selectivity have been observed between protic and aprotic solvents, suggesting that hydrogen bonding and proton transfer play important mechanistic roles [23]. These examples demonstrate the broader applicability of solvent-tuning strategies in various catalytic systems. However, such insights have rarely been extended to biomass-derived compounds like HMF, where selective hydrogenation can yield distinct products such as BHMF or DMF. More importantly, the fundamental mechanisms by which solvents regulate the reaction pathway and selectivity, especially through interfacial interactions with the catalyst and reactants, remain poorly understood. Therefore, a systematic investigation into the role of solvents in tuning HMF hydrogenation is essential for advancing both mechanistic understanding and practical control over product selectivity in biomass valorization.

In this work, a porous carbon-supported Ni-ZnO nanoparticles catalyst (denoted as Ni-ZnO/AC) was prepared by low-temperature coprecipitation method. The solvent effect of Ni-ZnO/AC catalyst in the selective hydrogenation of HMF was investigated employing hydrogen as the external hydrogen donor. In the polar aprotic/protic solvents, the polarity of the solvent influenced the product selectivity for the hydrogenation of HMF. Interestingly, the catalyst achieved high product selectivity for BHMF (97.5%) and DMF (99.5%) in HMF hydrogenation when using 1,4-dioxane and isopropanol (iPrOH) as solvents, respectively. The interaction between 1,4-dioxane/iPrOH solvent and catalyst can lead to different proton transfer processes and hydrodeoxygenation (HDO) behavior during hydrogenation of HMF. By integrating experimental results with reaction kinetics and density functional theory (DFT) calculations, the potential mechanism and solvent effect of Ni-ZnO/AC catalyst in the selective hydrogenation of HMF in the 1,4-dioxane/iPrOH solvent were elucidated. This study opens new avenues for the high-value utilization of furan chemicals by simply adjusting product selectivity through solvent variation in a single catalytic system.

## Experimental Section

### Materials

Coconut shell was supplied from Hainan Province of China. HMF (99%), 2-methyltetrahydrofuran (MeTHF, 99.5%) and zinc (II) nitrate hexahydrate (99%) were obtained from Aladdin Biochemical Technology Co., Ltd. BHMF (98%), 5-methylfurfural (MF, 98%), 5-methyl-2-furanmethanol (MFA, 97%), DMF (99%), 1-butanol (1-BuOH, 99%), 1-propanol (1-PrOH, 99%), methanol (MeOH, 99.5%) and sodium hydroxide (NaOH, 98%) were purchased from Macklin Biochemical Co., Ltd. Nickel (II) nitrate hexahydrate (99%) and 1,3-Dioxolane (99.9%) were supplied from Adamas Reagent Co., Ltd. Tetrahydrofuran (THF, 99.5%), 1,4-dioxane (99.5%), ethanol (EtOH, 95%), iPrOH (99.7%) and other reagents were purchased from Sinopharm Chemical Reagent Co., Ltd. All chemical reagents were used directly without further purification.

### Synthesis of Activated Carbon and Catalysts

#### Synthesis of Activated Carbon

Select dried coconut shells with a particle size of 4–5.6 mm as raw materials, weigh 20 g and place them in a tube furnace reactor. Set the heating rate to 10 °C min^−1^, control the water vapor flow rate at 1.3 g min^−1^, and activate the sample at 900 °C for 1 h. After activation, acid and water washing are carried out, and dried at 120 °C to obtain coconut shell activated carbon (AC).

#### Synthesis of Catalysts

Disperse 1 g of AC in 10 mL of water and sonicate for 30 min. Dissolve 0.51 g of Zn(NO_3_)_2_·6H_2_O and 0.55 g of Ni(NO_3_)_2_·6H_2_O in 10 mL of water, and then add the Zn-Ni nitrate mixture dropwise to AC under continuous stirring conditions. Next, place the above mixture in a low-temperature reactor (0 °C) and slowly add 0.1 M NaOH to facilitate the formation of metal hydroxide precipitates, simultaneously adjust the pH of the suspension to 9.5 and stir at room temperature for 2 h. Subsequently, filter the precipitates and wash them repeatedly with water until neutral. Dry the resulting sample in an oven at 105 °C. Following this, calcine the dried sample in an air atmosphere in an air atmosphere at 500 °C for 5 h. Finally, reduce the calcined sample in a 5% H_2_/N_2_ atmosphere at 500 °C for 2 h, named as Ni-ZnO/AC catalyst. Otherwise, Ni/AC and ZnO/AC were prepared using the same method.

The details of the catalyst characterization and other additional experimental methods are described in the Supporting Information.

## Results and Discussion

### Structural Characterizations of Ni-ZnO/AC

**Fig. 1:**
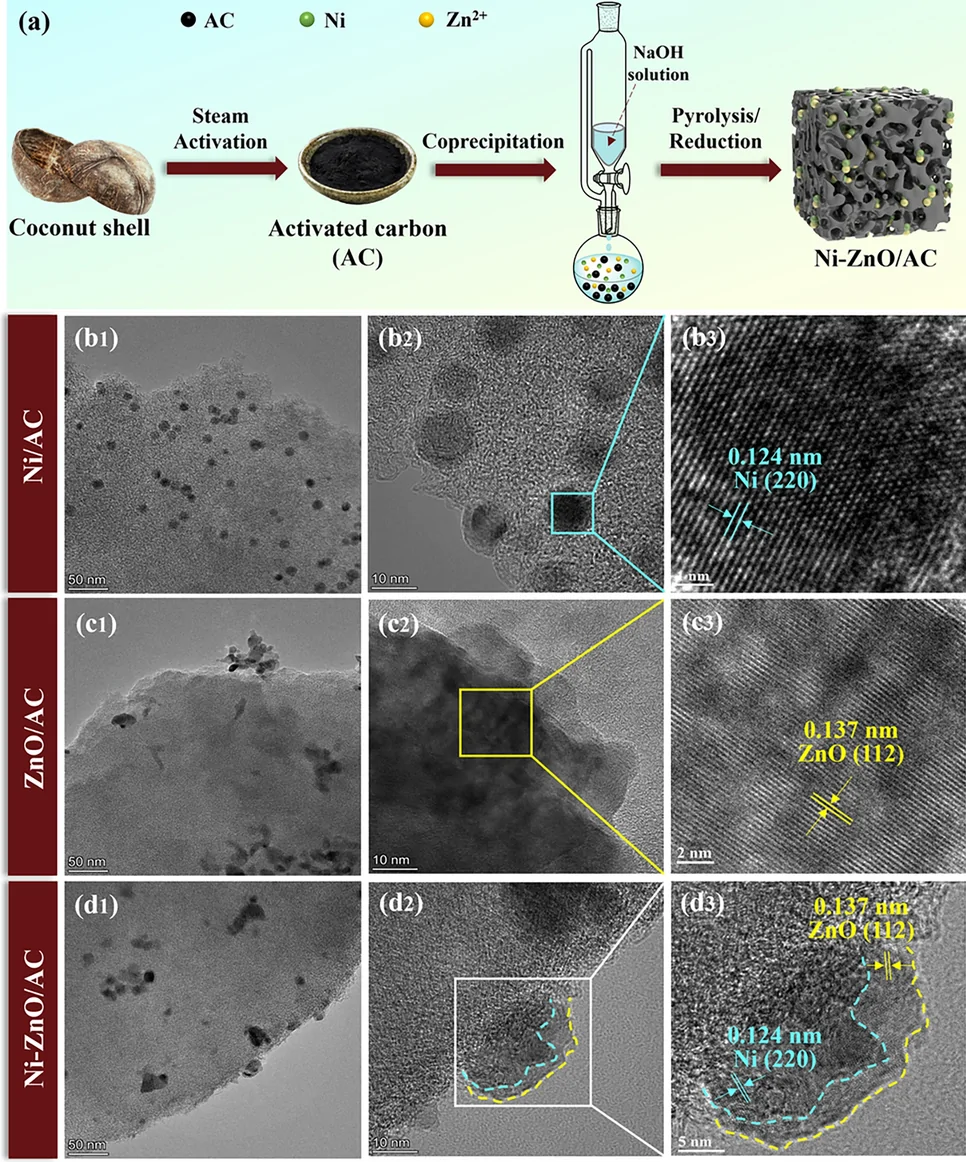
Synthesis process and microstructure characterization. **a** Synthesis procedures of Ni-ZnO/AC. TEM images of **b** Ni/AC, **c** ZnO/AC and **d** Ni-ZnO/AC

Coconut shell raw material, with its natural fiber skeleton, porous structure, and high carbon content advantages, has become an ideal precursor for the preparation of AC [24]. Through high-temperature treatment, the pore structure inside coconut shells further develops, and the specific surface area is significantly increased [25]. The adsorption capacity and mechanical stability of AC directly depend on this optimized pore foundation. Based on this, AC mainly composed of amorphous carbon can be produced from coconut shells using the steam activation method (Fig. S1). Its highly developed pore network not only endows the material with strong adsorption capacity, but also exhibits excellent stability due to the chemical inertness of the carbon skeleton. This combination of characteristics enables it to load active components efficiently, making it highly favored in the field of catalyst support [26]. Leveraging these advantageous characteristics, we successfully prepared porous carbon-supported Ni-ZnO nanoparticles catalyst by using low-temperature coprecipitation method, followed by pyrolysis and reduction treatment (Fig. 1a). The active components adhered firmly to the surface and within the pores of support, preserving the full accessibility of the pore structure (Fig. S1d). Inductively coupled plasma optical emission spectroscopy (ICP-OES) results indicate the contents of Ni and Zn are 10.09 wt% and 11.02 wt%, respectively (Table S1). Transmission electron microscopy (TEM) images reveal that the introduced Ni and Zn sources exist as metallic nickel and zinc oxide species, respectively. Metallic nickel appears as nanospheres with an average diameter of approximately 12.73 nm, while zinc oxide primarily exists as irregular nanoparticles (Figs. 1b, c and S2, S4, S5). The Ni/AC and ZnO/AC catalysts exhibit lattice spacing of 0.124 and 0.137 nm, corresponding to the lattice characteristics of Ni (220) and ZnO (112), respectively [27, 28]. Notably, the average particle size of Ni-ZnO/AC catalyst is approximately 15.19 nm (Fig. S3), which is slightly larger than that of Ni/AC. This increase is attributed to the partial encapsulation of Ni nanoparticles by ZnO domains, forming a semi-core–shell structure as illustrated in Fig. 1d. The presence of well-defined lattice fringes corresponding to Ni (220) and Zn (112) further confirms the coexistence of both crystalline phases. High-angle annular dark field scanning transmission electron microscopy (HAADF-STEM) images show a relatively consistent distribution of Ni and Zn elements (Fig. S6), indicating strong interfacial interaction between the two components. Such interaction can modulate the surface electronic structure and catalytic properties. Although the slightly larger particle size may reduce the specific area, the Ni-ZnO interface promotes charge transfer and enhances adsorption properties, thereby contributing to the improved catalytic activity and stability.

**Fig. 2:**
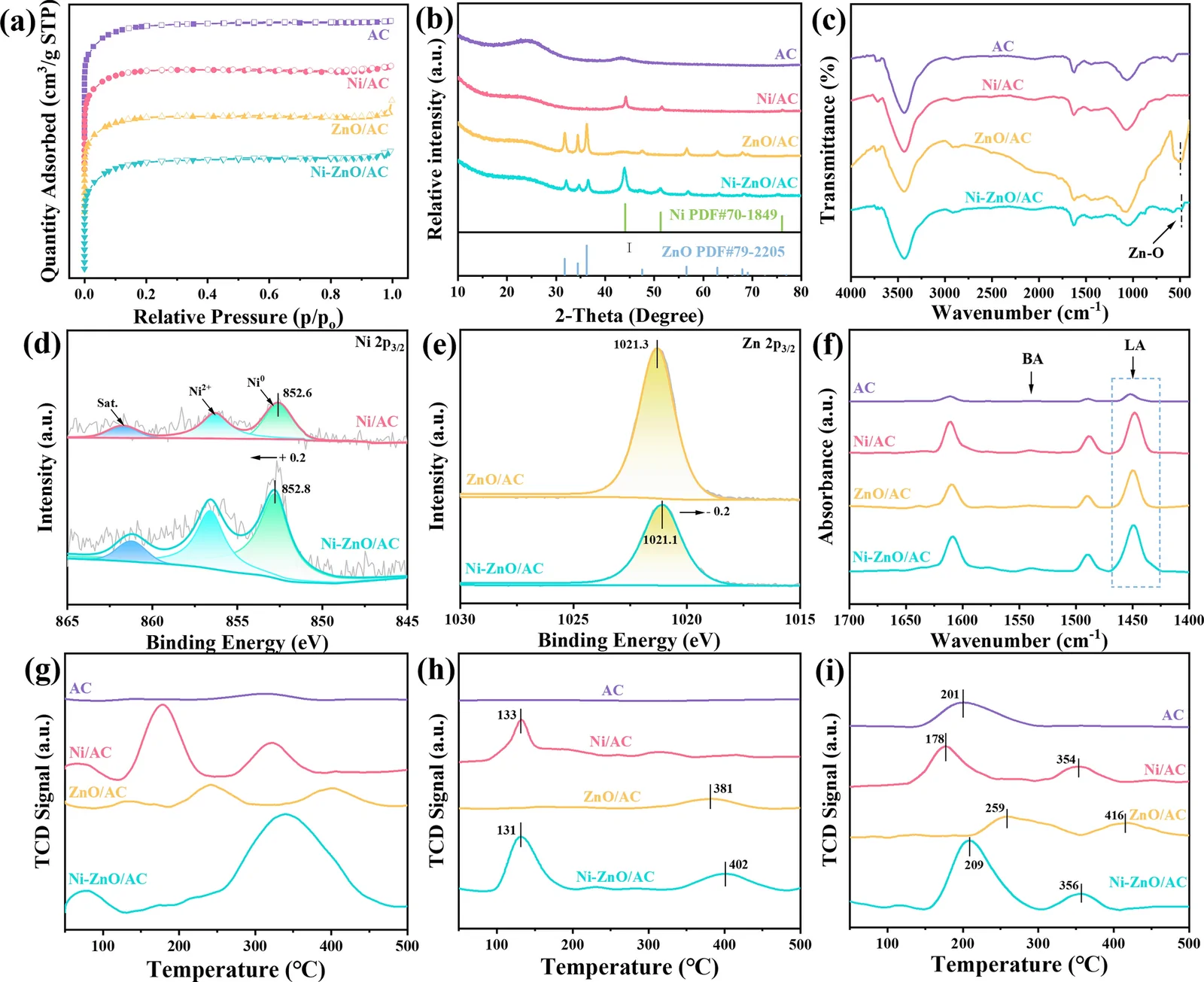
Comprehensive physicochemical characterization of various catalysts. **a** N_2_ adsorption/desorption isotherms, **b** XRD patterns, **c** FTIR spectra, **d** Ni 2*p*_3/2_, **e** Zn 2*p*_3/2_ XPS spectra, **f** Py-FTIR spectra, **g** NH_3_-TPD profiles, **h** H_2_-TPR profiles and **i** H_2_-TPD of various catalysts

The physicochemical properties of the catalysts, along with the interactions among their metal active sites, were characterized using various techniques. The N_2_ adsorption–desorption isotherms of AC, Ni/AC, ZnO/AC and Ni-ZnO/AC catalysts are presented in Fig. 2a. All catalysts display a typical type I isotherm, characterized by a rapid increase in adsorption at low pressures, suggesting that the catalysts possess a rich microporous structure and strong adsorption capacity [29]. According to the Brunauer–Emmett–Teller (BET) and non-local density functional theory (NLDFT) analyses, the specific surface area, total pore volume and micropore volume of the catalysts range from 1148 to 1270 m^2^ g^−1^, 0.64 to 0.74 cm^3^ g^−1^, and 0.43 to 0.49 cm^3^ g^−1^, respectively. The introduction of active components onto the support surface led to a reduction in specific surface area, total pore volume and micropore volume of Ni/AC, ZnO/AC and Ni-ZnO/AC catalysts (Table S1). X-ray diffraction (XRD) patterns, shown in Fig. 2b, verify that AC is amorphous, in accordance with the selected area electron diffraction (SEAD) result in Fig. S1c. The Ni/AC and ZnO/AC catalysts display distinct diffraction peaks corresponding to Ni (PDF#70–1849) and ZnO (PDF#79–2205), respectively. In contrast, the diffraction peaks of Ni-ZnO/AC catalyst align with the reference patterns of both Ni and ZnO [30]. Fourier transform infrared (FTIR) spectra of ZnO/AC and Ni-ZnO/AC catalysts reveal a clear Zn–O characteristic peak in ZnO/AC, while the corresponding peak becomes less prominent in Ni-ZnO/AC (Fig. 2c). This attenuation can be attributed to the possible interaction between Ni and ZnO, which may alter the local lattice environment of Zn–O bonds, weaken their vibrational intensity, or induce partial structural distortion. Additionally, surface coverage by Ni species and the presence of carbon support may obscure or overlap the Zn–O absorption, resulting in the reduced peak intensity observed [31]. The surface chemical states of Ni and ZnO species in the catalyst are analyzed via X-ray photoelectron spectroscopy (XPS), as demonstrated in Fig. 2d, e. The Ni 2*p*_3/2_ spectrum shows three characteristic peaks, one corresponding to the zero-valence state of Ni bonded to the support or metallic Ni, another due to the Ni^2+^ from nickel oxide or nickel hydroxide and a satellite peak [32]. The Ni 2*p*_3/2_ binding energy of Ni-ZnO/AC catalyst shifts upward by + 0.2 eV compared to Ni/AC catalyst, indicating a decrease in electron cloud density around the Ni. In the case of the Zn 2*p*_3/2_ spectrum of ZnO/AC catalyst, a single characteristic peak at 1021.3 eV is attributable to the Zn^2+^ of ZnO [33]. Interestingly, the Zn 2*p*_3/2_ of Ni-ZnO/AC catalyst shifts downward by − 0.2 eV compared with ZnO/AC catalyst, illustrating an increase in electron cloud density around the Zn species. The observed shifts in binding energies of Ni 2*p*_3/2_ and Zn 2*p*_3/2_ verify charge rearrangement and synergistic effect between Ni and ZnO species [34]. This complex interplay may enhance catalytic performance by facilitating electron transfer and improving reaction pathways in various chemical processes.

As is well known, the acidity and redox properties of catalyst play a pivotal role in the hydrogenation of HMF. The type and strength of acidity were characterized by pyridine-adsorbed Fourier transform infrared spectroscopy (Py-FTIR) and NH_3_ temperature programmed desorption (NH_3_-TPD), while the reducibility of the catalysts was evaluated by H_2_ temperature programmed reduction (H_2_-TPR). The Py-FTIR results, illustrated in Fig. 2f, exhibit a significant characteristic peak at 1450 cm^−1^ belonging to the Lewis acid (LA) sites [35], indicating that all four catalysts possess LA sites. However, no peaks are detected to the Brønsted acid (BA) sites across these catalysts. The strength of acidity was further tested through NH_3_-TPD (Fig. 2g), where the AC sample exhibited minimal acidity, likely attributed to residues from the acid washing process (Table S2). The desorption peak observed in the low-temperature range (< 110 °C) can be ascribed to these minor impurities on the catalyst surface. Ni-ZnO/AC catalyst displays a broad desorption curve between 200 and 450 °C, demonstrating the coexistence of moderate acid and strong acid sites [36]. Based on quantitative peak area fitting, the total acidity content of Ni-ZnO/AC catalyst is found to be the highest (1.21 μmol g^−1^, Table S2). To further elucidate the reduction performance of active sites on the support, H_2_-TPR results are depicted in Fig. 2h. Ni/AC catalyst exhibits a reduction peak appears at 133 °C, reflecting the partial reduction of nickel oxide or nickel hydroxide to metallic Ni. ZnO/AC catalyst shows a weak reduction peak at 381 °C, indicating the potential reduction trace Zn^2+^ species. In comparison, Ni-ZnO/AC catalyst not only displays a distinct reduction peak at 131 °C, but also a second peak at 402 °C. The higher-temperature peak suggests that Zn^2+^ may be substituted by Ni^2+^ at high temperatures, indicating an interaction between Ni and ZnO [37], which is consistent with the XPS results. Moreover, effective hydrogenation catalysts must not only activate and adsorb reaction substrates, but also achieve a suitable balance between the binding and release of H_2_ molecules [38]. The ability of catalysts to activate and dissociate H_2_ was evaluated by H_2_ temperature programmed desorption (H_2_-TPD). As shown in Fig. 2i, both Ni/AC and ZnO/AC catalysts reveal two desorption peaks in the range of 50–500 °C. Notably, Ni/AC catalyst has a lower desorption temperature alongside a larger peak area, revealing its superior capacity for activating and dissociating H_2_ compared to ZnO/AC catalyst. Furthermore, the interaction between Ni and ZnO in Ni-ZnO/AC catalyst result in a broad desorption peak between 150 and 300 °C, in addition to a desorption peak at 356 °C. It suggests that the synergistic action of Ni and ZnO enhances the ability to activate and dissociate H_2_, thereby providing a greater number of active H atoms for the hydrogenation [39].

### Catalytic Performance Evaluation toward Selective Hydrogenation of HMF

**Fig. 3:**
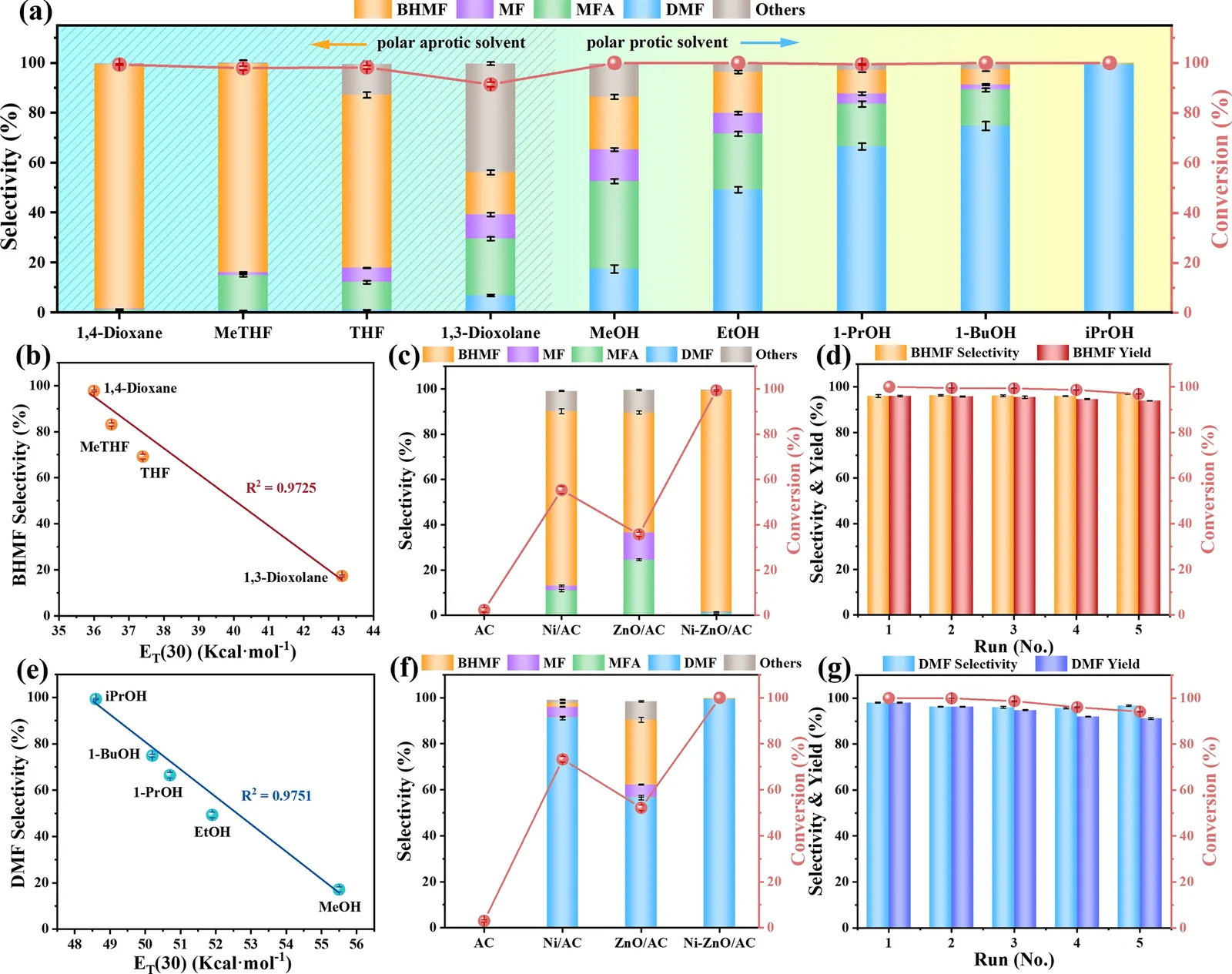
Effect of solvent type and polarity on catalytic hydrogenation of HMF and recyclability of Ni-ZnO/AC. **a** Influence of solvents on the HMF selective hydrogenation over Ni-ZnO/AC.** b** Relationship between polarity of polar aprotic solvent and BHMF selectivity. **c** HMF hydrogenation over different catalysts in the 1,4-dioxane solvent. **d** Recycling experiments of Ni-ZnO/AC in 1,4-dioxane solvent. **e** Relationship between polarity of polar protic solvent and DMF selectivity. **f** HMF hydrogenation over different catalysts in the iPrOH solvent. **g** Recycling experiments of Ni-ZnO/AC in iPrOH solvent. Reaction condition: 0.1 g HMF, 0.05 g catalyst, 1 MPa H_2_, 160 °C, 3 h

HMF, possessing multiple active function groups, can be hydrogenated to obtain various high value-added chemicals. Due to the highly oxygenated nature of feedstock, biomass upgrading reactions are normally conducted in a solvent [40]. This study investigates the solvent effect on the hydrogenation of HMF, focusing on polar aprotic solvents (1,4-dioxane, MeTHF, THF and 1,3-dioxolane) as well as polar protic solvents (MeOH, EtOH, 1-PrOH, 1-BuOH and iPrOH). Given that Ni-ZnO/AC catalyst exhibits superior acidity and reducibility, we prioritize the differences in the hydrogenation of HMF using Ni-ZnO/AC catalyst across different solvent systems (Fig. 3a). Experimental results indicate that the hydrogenation of HMF in polar aprotic solvents favors the formation of BHMF, with selectivity reaching 97.5% in 1,4-dioxane. Conversely, hydrogenation in polar protic solvents promotes the conversion to DMF, achieving a selectivity of up to 99.5% in iPrOH. By examining the polarity characteristics of the solvents, we establish a positive linear correlation between the polarity of solvents (E_T_(30)) and the selectivity of BHMF and DMF in polar aprotic and protic solvents, respectively (Fig. 3b, e). Specifically, higher solvent polarity results in enhanced substrate solubility within the catalytic system, facilitating more complete reactions [41]. Encouraged by these findings, AC, Ni/AC, ZnO/AC, and Ni_(x)_-ZnO_(y)_/AC catalysts with different Ni and Zn loadings are evaluated for the selective hydrogenation performance of HMF in 1,4-dioxane and iPrOH solvents (Fig. 3c, f, Table S3). Under the same reaction conditions, AC sample has no catalytic activity. Although Ni/AC and ZnO/AC catalysts display lower HMF conversion than the Ni-ZnO/AC catalyst in both two solvents, they still show selectivity for specific products, highlighting the decisive role of solvent in product selectivity. Ni_(x)_-ZnO_(y)_/AC catalysts with varying Ni and Zn loadings were prepared, and their performance in 1,4-dioxane and iPrOH solvents confirmed the above trends in BHMF and DMF selectivity. The catalyst with actual Ni and Zn contents of 10.09 and 11.02 wt%, respectively, exhibited the best performance for the selective hydrogenation of HMF. Ni serves as the primary hydrogenation site, determining overall activity, while ZnO modulates electronic structure to enhance selectivity. Their synergistic interaction, especially at balanced loadings, significantly improves both conversion and product distribution, outperforming systems with only high Ni or Zn content. Subsequently, a detailed investigation is conducted on HMF hydrogenation involving the Ni-ZnO/AC catalyst in the two solvents (Figs. S7 and S8). Figure S9 illustrates the relationship between catalytic performance and reaction conditions, including reaction temperature, time and H_2_ pressure. In 1,4-dioxane, both HMF conversion and BHMF selectivity initially increase with rising reaction temperature, stabilizing afterward. However, with prolonged reaction time and increased H₂ pressure, BHMF selectivity first rises and then declines, attributed to ongoing reaction conversion. The optimal conditions for the hydrogenation of HMF to BHMF in this solvent are determined to be 160 °C, 3 h, and 1 MPa H₂. In iPrOH, DMF selectivity initially increases with higher reaction temperature and duration, peaking at 160 °C and 3 h with a maximum value of 99.5%. Notably, HMF conversion remains low at 13.6% when H₂ pressure is 0. However, when H₂ pressure is elevated to 0.5 MPa, HMF conversion rapidly rises to 99.6%, with a DMF selectivity of 48.2%. A further increase in H₂ pressure to 1 MPa results in complete HMF conversion and 99.5% DMF selectivity, indicating that H₂ acts as the primary hydrogen donor in the hydrogenation of HMF to DMF in iPrOH. Additionally, the recyclability of Ni-ZnO/AC catalyst in both solvents is also investigated (Fig. 3d, g). After five consecutive cycles, both HMF conversion and corresponding products yield/selectivity demonstrate only slight decreases (< 8%). Characterization of the recovered catalyst reveals minimal losses in Ni (0.20 wt%) and Zn (0.33 wt%) content, while the morphological characteristics and physicochemical properties remain largely unchanged. It indicates that Ni-ZnO/AC catalyst maintains satisfactory stability after five consecutive cycles (Table S1, Figs. S10 and S11).

**Fig. 4:**
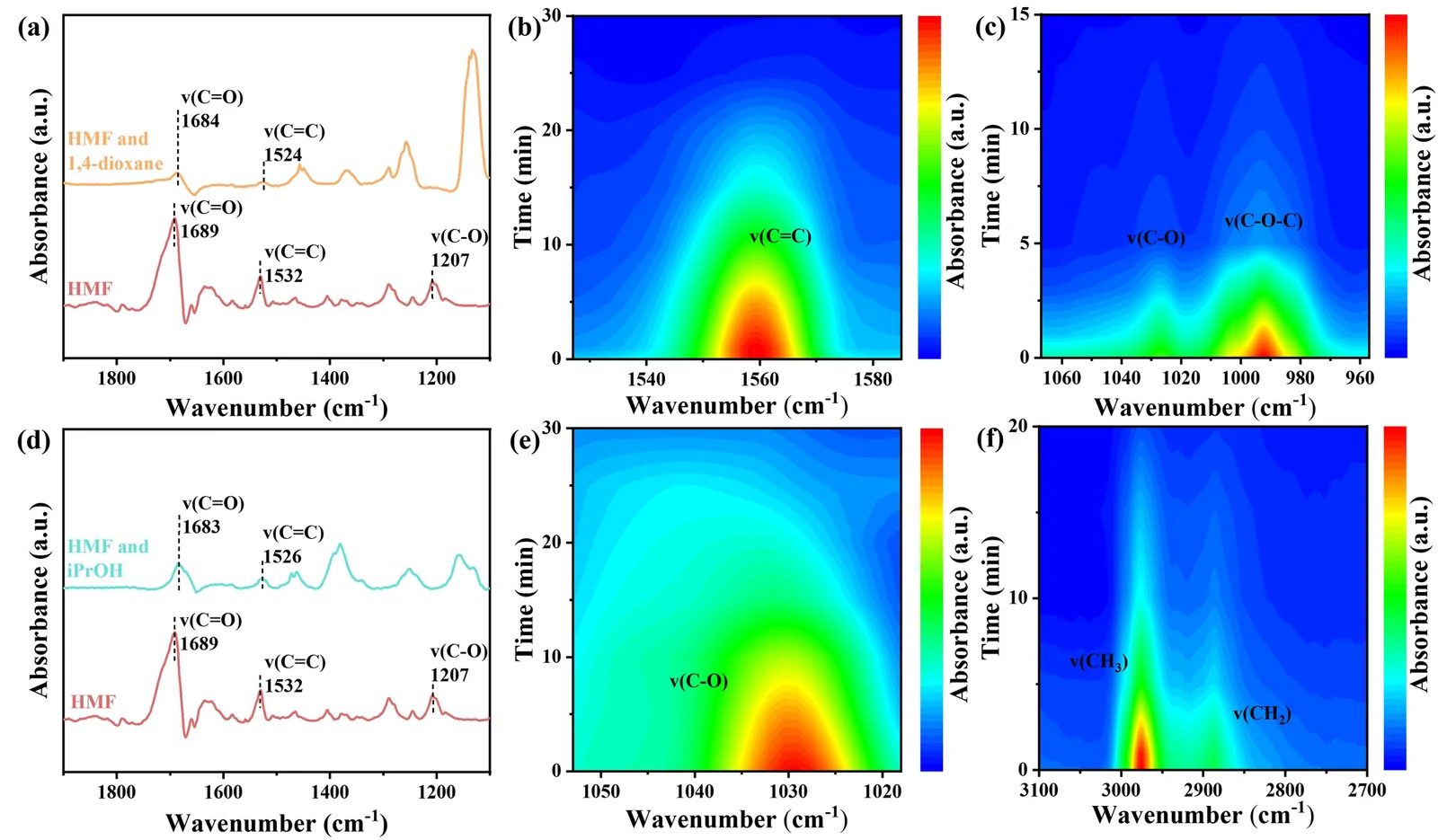
In situ FTIR studies on solvent effects during HMF hydrogenation over Ni-ZnO/AC. Studies on the solvent effect in the presence of Ni-ZnO/AC catalyst. In situ FTIR spectra of HMF adsorption on Ni-ZnO/AC catalyst acquired within 1900–1100 cm^−1^ through introducing **a** 1,4-dioxane and **d** iPrOH. In situ FTIR spectra of hydrogenation of BHMF over Ni-ZnO/AC catalyst introducing **b-c** 1,4-dioxane and **e–f** iPrOH solvents with H_2_ as a reaction gas

In addition, in situ Fourier transform infrared spectroscopy (in situ FTIR) was employed to monitor the changes in functional groups of HMF in the 1,4-dioxane and iPrOH solvents under the influence of the Ni-ZnO/AC catalyst. The in situ FTIR spectrum of pure HMF, shown in Fig. 4a, d, reveals characteristic peaks at 1689, 1532, and 1207 cm^−1^, corresponding to v(C = O), v(C = C), and v(C-O), respectively [42]. In both the 1,4-dioxane and iPrOH solvents, weak red shifts are observed for the v(C = O) and v(C = C) vibrations of HMF, indicating that the catalyst surface exhibits enhanced adsorption activity for C = O and C = C groups in the presence of solvents. Due to the constrained state of bridge/multi-coordination adsorption, specific functional groups lose their corresponding infrared signals during chemical adsorption on the catalyst surface [43]. BHMF, a key intermediate in the hydrogenation of HMF, in situ FTIR spectroscopy was investigated to understand dynamic catalytic reaction process of BHMF on the Ni-ZnO/AC catalyst with the presence of 1,4-dioxane and iPrOH. The in situ FTIR spectrum of BHMF reveals three characteristic peaks at 1560, 1029, and 1001 cm^−1^, corresponding to v(C = C), v(C-O), and v(C–O–C), respectively (Fig. S12) [44]. In the case of BHMF with 1,4-dioxane system (Fig. 4b, c), the C-O bond of BHMF exhibits a significant red-shift compared to that of pure BHMF, suggesting substantial adsorption of BHMF on the catalyst surface. Following the continuous hydrogen flow, both the C-O and C = C signals gradually diminish and completely disappear within 30 min, verifying that BHMF molecules desorb from the catalyst surface rather than participating in further reactions. In contrast, as for BHMF with iPrOH system (Fig. 4e, f), the C-O signal decreases rapidly upon hydrogen introduction; however, a new methyl-related band emerges at 2975 cm^−1^, confirming significant cleavage of C-O bond by the Ni-ZnO/AC catalyst in the iPrOH solvent. This finding aligns with the observed optimal selectivity toward DMF.

### Theoretical Assessment for Solvent-Regulated Selective Hydrogenation of HMF

**Fig. 5:**
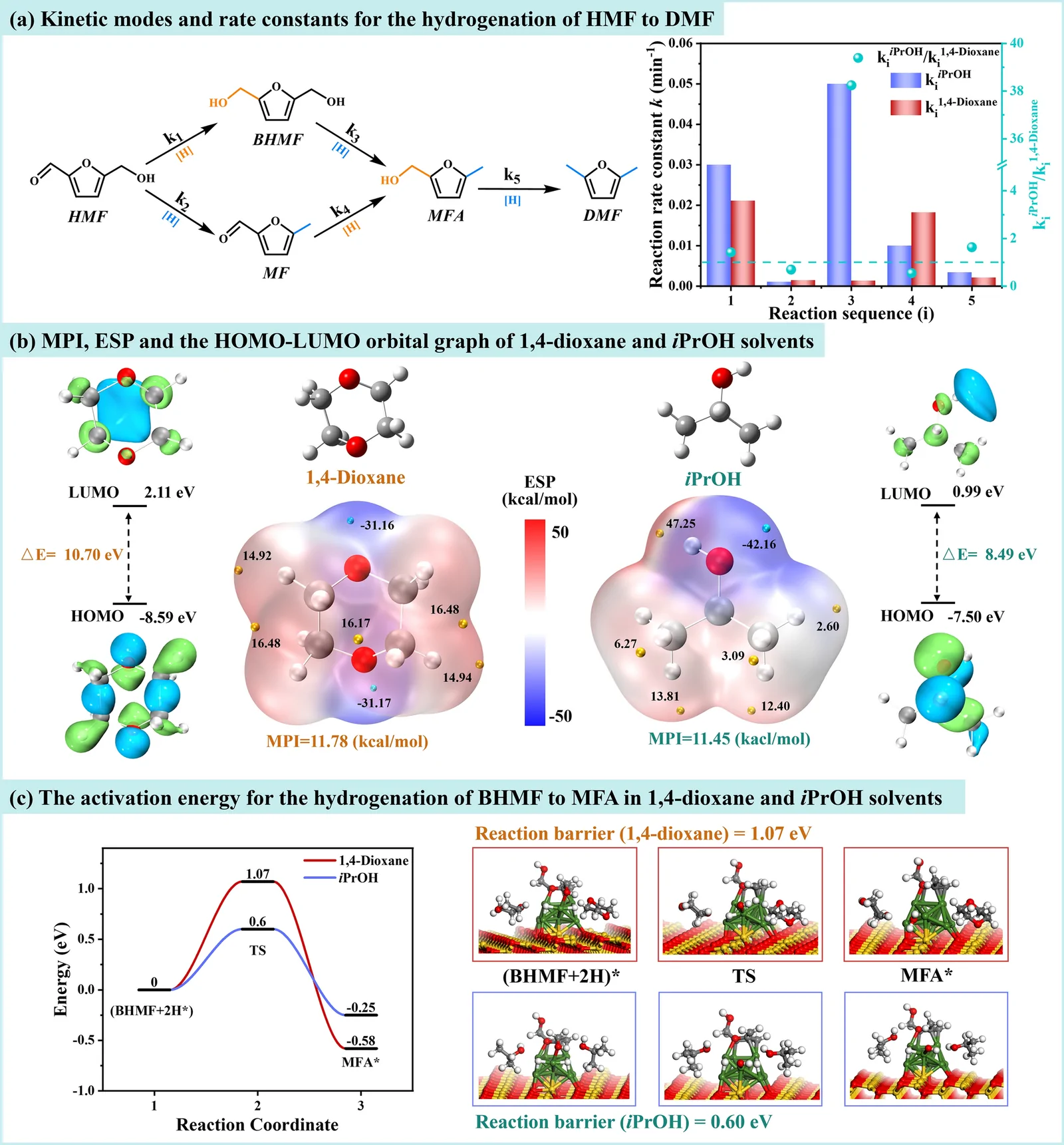
Mechanistic insights into HMF hydrogenation in different solvents. **a** Kinetic modes and rate constants for the hydrogenation of HMF to DMF. **b** MPI, ESP and the HOMO–LUMO orbital graph of 1,4-dioxane and iPrOH solvents. **c** The activation energy for the hydrogenation of BHMF to MFA in 1,4-dioxane and iPrOH solvents. (Ni: green, Zn: yellow, C: gray black, O: red, H: white)

According to the above experimental results and previous work, the kinetic behavior for the hydrogenation of HMF to DMF follows pseudo-first-order reaction kinetics [45, 46]. The reaction pathway diagram is depicted in Fig. 5a. For the initial conversion, the formation rate of BHMF from HMF in iPrOH solvent is higher than that in 1,4-dioxane solvent (k_1_^iPrOH^/k_1_^1,4−Dioxane^ = 1.5), indicating that Ni-ZnO/AC catalyst has more outstanding hydrogenation behavior for the C = O bond of HMF in iPrOH. In contrast, the reaction rate constant (k_2_) for the hydrogenation of HMF to MF is much lower than in the other reaction steps, highlighting this process as the rate-determining step. The markedly greater value of k_1_ compared to k_2_ further underscores the ease of converting HMF into BHMF. A significant difference in the hydrogenation of BHMF to MFA is observed between the two solvents, with k_3_^iPrOH^ being 38.2 times greater than k_3_^1,4−Dioxane^, indicating a substantial disparity in the HDO capabilities. The extremely low value of k_3_^1,4−Dioxane^ suggests that the HDO process in this solvent is nearly stagnant, rendering BHMF quite stable. Thus, HMF hydrogenation exhibits higher selectivity for BHMF in the presence of the Ni-ZnO/AC catalyst and 1,4-dioxane. It is also found that k_3_^iPrOH^ > k_3_^1,4−Dioxane^ and k_5_^iPrOH^ > k_5_^1,4−Dioxane^, illustrating that the catalyst has a more prominent HDO ability in iPrOH solvent, which elucidates that the interaction of Ni-ZnO/AC catalyst and iPrOH has a higher selectivity of DMF.

To further elucidate the characteristics of two solvents, molecular polarity index (MPI) and molecules electrostatic potential (ESP) were calculated, as presented in Fig. 5b. A higher MPI value indicates greater molecular polarity and a stronger ability to interact with BHMF through electrostatic forces. The minimum electrostatic potential point (represented by a blue sphere) of the solvent molecules is situated near the O atom, suggesting that this area is conducive to electrostatic interactions with BHMF, thereby enhancing the solvent effect. The fundamental distinction between polar aprotic and polar protic solvents lies in whether they contain protons and self-dissociate behavior. Polar aprotic solvents lack hydrogen bond donors and function only as hydrogen bond acceptors, while polar protic solvents possess both types of hydrogen bonding functionalities [47]. The absence of hydrogen bond donors in 1,4-dioxane hinders the continuous HDO of BHMF. Specially, the MPI value of 1,4-dioxane is slightly greater than that of iPrOH, revealing a stronger binding affinity between 1,4-dioxane and BHMF during hydrogenation, suggesting that the substrate and intermediate products exhibit varying stabilization effects in the two solvents. Moreover, to evaluate the electronic property of solvents, the highest occupied molecular orbital (HOMO) and the lowest unoccupied molecular orbital (LUMO) of 1,4-dioxane and iPrOH were computationally determined, and the HOMO–LUMO energy gap (ΔE) was calculated, as illustrated in Fig. 5b. A smaller ΔE generally reflects enhanced molecular polarizability and a greater tendency for charge redistribution, implying lower excitation energy and increased electronic responsiveness [48]. Compared to 1,4-dioxane, iPrOH exhibits a smaller ΔE, indicating stronger polarizability and greater potential for electronic interaction with the catalyst surface and adsorbed intermediates. Furthermore, as a protic solvent, iPrOH can donate protons and form hydrogen bonds with reactants and transition-state species, thereby stabilizing key intermediates and facilitating a hydrogen shuttle mechanism that assists in hydroxyl group departure during the hydrogenolysis step. In contrast, 1,4-dioxane, being aprotic and less polarizable, lacks the capacity for such interactions. Therefore, the smaller ΔE of iPrOH is consistent with its enhanced ability to mediate interfacial charge transfer and proton-coupled processes that are critical for effective hydrogenation behavior.

Based on the reaction kinetic model, it is evident that the reaction rate constants (k_3_) for the hydrogenation of BHMF to MFA differ significantly between the two solvents. Consequently, only the reaction barrier of the critical step was calculated, and the results are depicted in Fig. 5c. A simplified model of Ni-ZnO/AC catalyst was constructed for calculation (Fig. S13), and the hydrogenation step was simulated while considering explicit solvent molecules. The hydrogen molecule readily dissociates on the ZnO supported Ni cluster, leading to the formation of surface-adsorbed hydrogen (*H). Subsequently, *H attacks BHMF, resulting in the formation of MFA and the migration of hydroxyl to Ni surface. In the system containing two 1,4-dioxane molecules, the activation barrier for the hydrogenation step is 1.07 eV. Conversely, as the protic iPrOH molecules are involved in the reaction, the barrier is 0.60 eV, significantly lower than that observed in 1,4-dioxane. Apparent hydrogen shuttle effect significantly facilitates the reducing process, in which the proton in iPrOH migrates to BHMF, promoting the leaving of hydroxyl group. Simultaneously, the alkaline iPrO- anion captures *H on the Ni surface, regenerating the solvent molecule (Fig. S14). Thus, the HDO process is more readily facilitated in iPrOH. These observations prompted us to further evaluate the relative contributions of solvent polarity and proton-donating ability toward product selectivity. Overall, while a positive correlation is observed between solvent polarity (E_T_(30)) and product selectivity within both polar aprotic/protic solvent classes (Fig. 3b, e), mechanistic investigations reveal that proton-donating ability plays a more decisive role in governing the final reaction pathway. Specifically, polar protic solvents like iPrOH not only enhance solubility and substrate–catalyst contact due to their polarity, but also participate directly in the hydrogenolysis process through proton transfer, as evidenced by their lower ΔE, higher polarizability, and ability to form hydrogen bonding networks. In contrast, polar aprotic solvents such as 1,4-dioxane, despite having comparable or even slightly higher MPI values, lack proton-donating capacity and thus cannot effectively promote the HDO step from BHMF to DMF. This explains why polarity alone does not fully account for the observed product selectivity, and highlights proton transfer as the critical mechanistic driver behind solvent-regulated hydrogenation behavior.

## Conclusions

In this contribution, we report the synthesis of porous carbon-supported Ni-ZnO nanoparticles catalyst (Ni-ZnO/AC) via low-temperature coprecipitation, which shows exceptional performance for the selective hydrogenation of HMF to high value-added products, achieving a remarkable selectivity for BHMF (97.5%) and DMF (99.5%) when using 1,4-dioxane and iPrOH as solvents, respectively. Both experimental results and theoretical calculations confirm that the solvent-catalyst interaction jointly regulates the hydrogenation pathway by influencing not only the reaction rate, but also the selectivity toward different products. Notably, this study demonstrates a scalable catalytic strategy, which allows tunable control over product distribution by simply altering the solvent environment. This solvent-guided selectivity within a single catalytic system shows promising potential for industrial application, as BHMF and DMF are valuable intermediates for biomass-based materials and fuels. Therefore, this work provides valuable insights into biomass conversion chemistry and offers a practical route for the sustainable and tunable valorization of HMF and other biomass-derived platform molecules.

## Supplementary Information

Below is the link to the electronic supplementary material.

Supplementary file1 (DOCX 2535 KB)
