# Supplementary material for: A Promising Strategy for Solvent-Regulated Selective Hydrogenation of 5-Hydroxymethylfurfural over Porous Carbon-Supported Ni-ZnO Nanoparticles
Source: Nanomicro Lett. 2025 Jul 18;18:5. doi: 10.1007/s40820-025-01847-5 (PMC12274185; doi:10.1007/s40820-025-01847-5)
Supplement: Supplementary file 1 — Supplementary file1 (DOCX 2535 KB) [file 40820_2025_1847_MOESM1_ESM.docx]

Supporting Information for

**A Promising Strategy for Solvent-Regulated Selective Hydrogenation of 5-Hydroxymethylfurfural over** **Porous Carbon-Supported Ni-ZnO Nanoparticles**

Rulu Huang ^1, 2^, Chao Liu ^3^, Kaili Zhang ^2^, Jianchun Jiang ^1, 2,^ *, Ziqi Tian ^4,^ *, Yongming Chai ^5,^ *, Kui Wang ^1, 2,^ *

^1^ Institute of Chemical Industry of Forest Products, Chinese Academy of Forestry, Biomass Energy and Material Key Laboratory of Jiangsu Province, Nanjing 210042, P. R. China

^2^ Co-Innovation Center of Efficient Processing and Utilization of Forest Resources, Nanjing Forestry University, Nanjing 210037, P. R. China

^3^ School of Materials Science and Engineering, Central South University of Forestry and Technology, Changsha 410004, P. R. China

^4^ Ningbo Institute of Materials Technology and Engineering, Chinese Academy of Sciences, Ningbo 315201, P. R. China

^5^ State Key Laboratory of Heavy Oil Processing, College of Chemistry and Chemical Engineering, China University of Petroleum (East China), Qingdao 266580, P. R. China

*Corresponding authors. E-mail: [jiangjc@icifp.cn](mailto:jiangjc@icifp.cn) (Jianchun Jiang); [tianziqi@nimte.ac.cn](mailto:tianziqi@nimte.ac.cn) (Ziqi Tian); [ymchai@upc.edu.cn](mailto:ymchai@upc.edu.cn) (Yongming Chai); [wangkui@caf.ac.cn](mailto:wangkui@caf.ac.cn) (Kui Wang)

**S1 Experimental Procedures**

**S1.1 Catalyst Characterization**

A scanning electron microscope (SEM, ZEISS GeminiSEM 300) was used to observe the morphological characteristics of catalysts. In addition, transmission electron microscopy (TEM, FEI Talos F200X G2) at an accelerating voltage of 100 kV was used to observe the dispersion of metal nanoparticles on the catalyst surface. The specific surface area, pore volume, and average pore size of the catalyst were measured using a physical adsorption instrument (Micromeritics ASAP 2460). The catalysts were vacuumed at 300 °C for 6 h before measurement, followed by N_2_ adsorption-desorption experiments at -196 °C. The specific surface area and pore structure parameter distribution were calculated by the Brunnauer Emmett Teller (BET) and Non-local Density Functional Theory (NLDFT) methods, respectively. After acid dissolution pretreatment of the catalysts, the elemental contents in the catalysts were analyzed by the inductively coupled plasma optical emission spectroscopy (ICP-OES, Thermo Fisher iCAP PRO). X-ray diffraction (XRD) measurements of catalysts were conducted on an X-ray diffractometer (Rigaku SmartLab SE) operated at 40 kV and 40 mA with Cu Ka radiation source over a 2θ range of 5 to 90° at a scanning speed of 5 °/min. Fourier transform infrared (FTIR) spectrum was recorded on a Nicolet iS50 spectrometer using the standard KBr pellet method. X-ray photoelectron spectroscopy (XPS) profiles were conducted on an X-ray photoelectron spectrometer (Thermo Scientific K-Alpha) with an Al Kα X-ray source (hν = 1486.6 eV) to test the surface element composition of catalysts and valence distribution of metal species. The acid type and concentration of catalysts were determined by pyridine-adsorbed Fourier transform infrared spectroscopy (Py-FTIR, Bruker Tensor 27). In Py-FTIR spectra, the characteristic peaks located at 1450 cm^-1^ and 1545 cm^-1^ were attributed to Lewis acid (LA) and Brønsted acid (BA) sites, respectively. The acidic strength and content of samples were characterized by NH_3_ temperature programmed desorption (NH_3_-TPD) on a Micromeritics AutoChem II 2920 adsorption instrument. Before the adsorption, the sample (ca. 50 mg) was activated at 120 ºC with a He flow rate of 30 mL/min for 1 h. After cooling down to 50 ºC, the NH_3_-He (10%-90%) mixture was dosed for 1 h, followed by sweeping under He. The system temperature was then increased from 50 to 500 ºC with a heating rate of 10 ºC/min. The NH_3_ desorption signals were detected by a TCD detector. H_2_ temperature programmed reduction (H_2_-TPR) was carried out on a Micromeritics AutoChem II 2920 apparatus to analyze the interaction between metal and carrier. H_2_ temperature programmed desorption (H_2_-TPD) was used to explore the interaction between the catalysts and H_2_ (10% H_2_/Ar) on a temperature-programmed chemical adsorption instrument (Micromeritics AutoChem II 2920). The system temperature detection range was from 50 ºC to 500 ºC with a heating rate of 10 ºC/min. The H_2_ desorption signals were recorded by a thermal conductivity detector. In-situ Fourier-transform infrared spectroscopy (in-situ FTIR) measurement was performed on a Nicolet iS50 spectrometer equipped with a mercury cadmium telluride (MCT) detector. Firstly, each sample was pre-reduced at 500 ºC with H_2_ flow for 1 h, and cooled to reaction temperature in the H_2_ flow. Afterward, 10 μL of the solution (1 mg of HMF/BHMF, 10 μL of solvent) was introduced to the sample, and the cell was purged with H_2_ and sealed at 0.1 MPa H_2_ in the end. Subsequently, the in-situ FTIR spectra of the hydrogenation at different times were recorded.

**S1.2 Rection Procedures**

The hydrogenation reaction was carried out in a 50 mL cylindrical stainless-steel reactor (Yanzheng Experimental Instrument Co., Ltd., China) equipped with a thermocouple-controlled temperature and magnetic stirrer. A typical experimental operation was as follows: a mixture of catalyst, substrate and solvent was loaded into the reactor. Next, sealed the reactor and used H_2_ to blow the reactor three times. Then, pressurized the reactor to a specific value at room temperature and heated it according to the set temperature program while stirring at 600 rpm. After the reaction, take out the reactor and quickly put it into a cold-water bath for cooling to terminate the reaction. Subsequently, the cooled mixture was filtered to collect the filtrate for product analysis and the separated catalyst by centrifugation was washed with deionized water three times. Finally, the catalyst was dried in a vacuum at 80 °C for 12 h, and the recovered catalyst was directly used in the next cycle under the same reaction conditions.

**S1.3 Product Analysis**

Accurate qualitative analysis of the reaction products was carried out by gas chromatography-mass spectrometer (GC-MS, Agilent 8890-7000D) equipped with an HP-5 MS column (30 m × 0.25 mm × 0.25 μm). In addition, a gas chromatograph (GC, Shimadzu 2010 plus) equipped with a flame ionization detector (FID) and a HP-5 column (30 m × 0.32 mm × 0.25 μm) was used for subsequent quantitative analysis of the product. Both GC-MS and GC analysis used the following detection heating procedure: set the injection port and detector temperature to 280 °C, with an initial temperature of 40 °C for 2 min, followed by a temperature rise to 110 °C at a heating rate of 10 °C/min, then to 120 °C at a heating rate of 1 °C/min, followed by heating at a rate of 20 °C/min to 250 °C and maintain temperature for 1 min. The concentrations of substrate and products were calculated based on the external standard method. The conversion of substrate (*C*, %), yield (*Y*, %) and selectivity (*S*, %) were calculated according to Eqs. (S1) - (S3):

$C=\left( 1-n_{f}/n_{i} \right)\times100\%$ (S1)

$Y=\frac{n_{product}}{n_{i}}\times100\%$ (S2)

$$S=\frac{Y}{C}\times100\% (S3)$$

Where, $n_{i}$ was the moles of the added substrate, $n_{f}$ accounted for the moles of the remained substrate in the post-treated liquid, and $n_{product}$ was the moles of product in the post-treated liquid.

**S2 Computational Details**

**S2.1 Kinetic Models for the hydrogenation of HMF to DMF**

The kinetic behaviors were expected to follow pseudo-first-order kinetics for all reaction steps (Fig. 5a). Here, k_i_ represents the reaction rate constant for each step, where i denotes the order of the reaction (i = 1, 2, 3, 4, 5).

$$\frac{dc_{HMF}}{dt}=-\left（ k_{1}+k_{2} \right）c_{HMF} \left( S4 \right)$$

$\frac{dc_{BHMF}}{dt}=k_{1}\cdot c_{HMF}-k_{3}\cdot c_{BHMF}$ (S5)

$\frac{dc_{MF}}{dt}=k_{2}\cdot c_{HMF}-k_{4}\cdot c_{MF}$ (S6)

$\frac{dc_{MFA}}{dt}=k_{3}\cdot c_{BHMF}+k_{4}\cdot c_{MF}-k_{5}\cdot c_{MFA}$ (S7)

$\frac{dc_{DMF}}{dt}=k_{5}\cdot c_{MFA}$ (S8)

The reaction rate constants for the hydrogenation of HMF to DMF in 1,4-dioxane and iPrOH over Ni-ZnO/AC catalyst can be derived by fitting the experimental data shown in Fig. S7c, d, respectively. The integration of the aforementioned formulae is presented as follows:

$c_{HMF}=c_{HMF0}e^{-(k_{1}+k_{2})t} (S9)$

$c_{BHMF}=c_{HMF0}k_{1}\frac{e^{-k_{3}t}-e^{-{(k_{1}+k}_{2})t}}{k_{1}+k_{2}-k_{3}} (S10)$

$c_{MF}=c_{HMF0}k_{2}\frac{e^{-k_{4}t}-e^{-{(k_{1}+k}_{2})t}}{k_{1}+k_{2}-k_{4}} (S11)$

$$c_{MFA}=c_{HMF0}k_{2}k_{4}\left[ \frac{e^{-k_{2}t}}{\left( k_{4}-k_{2} \right)\left( k_{5}-k_{2} \right)}-\frac{e^{-k_{4}t}}{\left( k_{2}-k_{4} \right)\left( k_{5}-k_{4} \right)}+\frac{e^{-k_{5}t}}{\left( k_{2}-k_{5} \right)\left( k_{4}-k_{5} \right)} \right] \left( S12 \right)$$

$$c_{DMF}=c_{HMF0}\left[ \frac{k_{4}k_{5}\left( {1- e}^{-k_{2}t} \right)}{\left( k_{4}-k_{2} \right)\left( k_{5}-k_{2} \right)}-\frac{k_{2}k_{5}\left( {1- e}^{-k_{4}t} \right)}{\left( k_{2}-k_{4} \right)\left( k_{5}-k_{4} \right)}+\frac{k_{2}k_{4}\left( {1- e}^{-k_{5}t} \right)}{\left( k_{2}-k_{5} \right)\left( k_{4}-k_{5} \right)} \right] (S13)$$

Where c_HMF0_ is the initial concentration of HMF.

**S2.2 Wave function analysis**

The wave function information of solvent molecules was calculated by Gaussian 09 and GaussView 5.0 software packages [S1, S2]. The geometric structures of 1,4-dioxane and iPrOH were fully optimized by using B3LYP/6-31G* and grime's DFT-D3(BJ) empirical dispersion correction, and no imaginary frequencies were found in the optimized structures by frequency calculation [S3]. Multiwfn analysis program was used for wave function analysis [S4], the results of electrostatic potential and molecular orbital analysis were visualized using VMD (1.9.3) software [S5].

**S2.3 Quantum chemistry theoretical calculations**

To construct the ZnO supported Ni cluster model, a three-layered ZnO (100) slab within *p*(3 × 5) periodic cells was employed as the substrate. A vacuum layer with thickness of 25 Å was applied to eliminate periodic interactions between neighboring images. The Ni cluster consisting of 13 atoms was placed on the ZnO slab, where 9 atoms were in the bottom layer contacting with the substrate, and the remaining 4 atoms were positioned in the top layer. In the top layer of the Ni cluster, the central atom is surrounded by 3 atoms. To simulate the hydrogenation step in various solvents, two explicit solvent molecules were placed close to the reaction site.

Spin-polarized DFT calculations were performed using the Vienna Ab initio Simulation Package (VASP 5.4.4) [S6], employing the projector augmented wave (PAW) method to describe the core electrons [S7]. The Kohn-Sham equations were solved using the generalized gradient approximation (GGA) with the Perdew-Burke-Ernzerhoff (PBE) functional [S8]. The DFT-D3 method of Grimme with zero-damping function was implemented to account for van der Waals interactions [S9]. The valence electronic structures were expanded on a plane wave with a cutoff energy of 450 eV. Since the model contains hundreds of atoms, all the calculations only sampled the Gamma point. The convergence criteria were set to 10^-4^ eV for electronic energy and 0.05 eV Å⁻¹ for force. A *U* value of 3.0 eV was applied to describe the *d*-electrons of Ni. Transition states were located using the Climbing Image Nudged Elastic Band (CI-NEB) method with a force convergence criterion of 0.3 eV Å⁻¹ [S10].

**S3 Supplementary Figures and Tables**


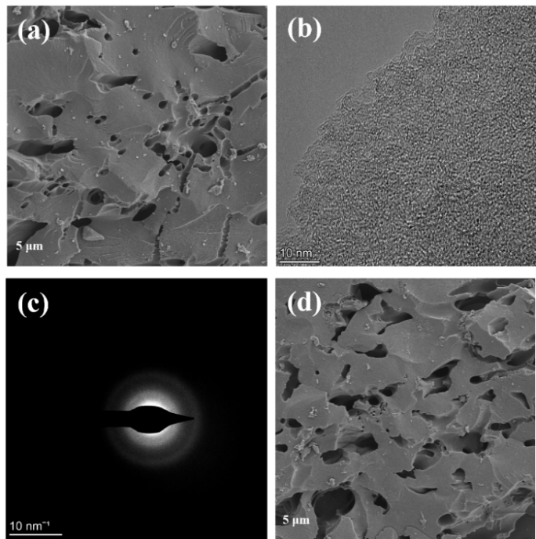


**Fig. S1** **a** SEM, **b** TEM and **c** SAED images of AC. **d** SEM image of Ni-ZnO/AC

**Fig. S2** Particle size distribution of Ni/AC

**Fig. S3** Particle size distribution of Ni-ZnO/AC


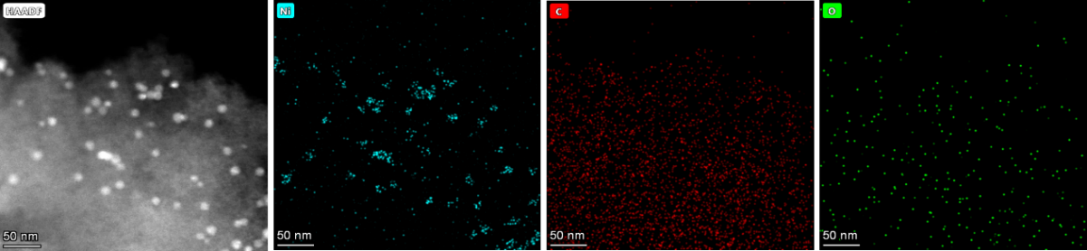


**Fig. S4** HAADF-STEM images of Ni/AC


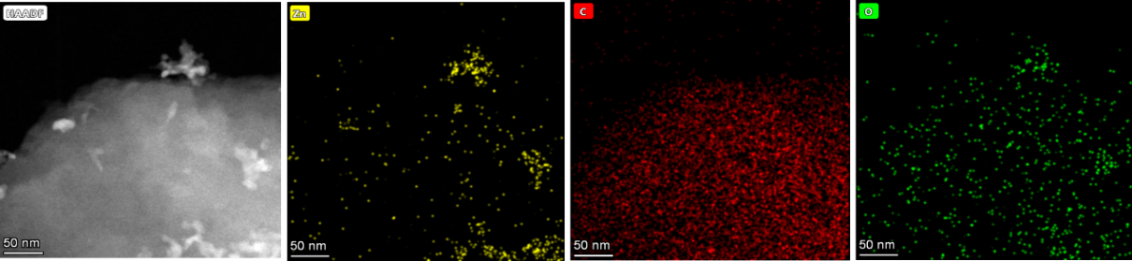


**Fig. S5** HAADF-STEM images of ZnO/AC


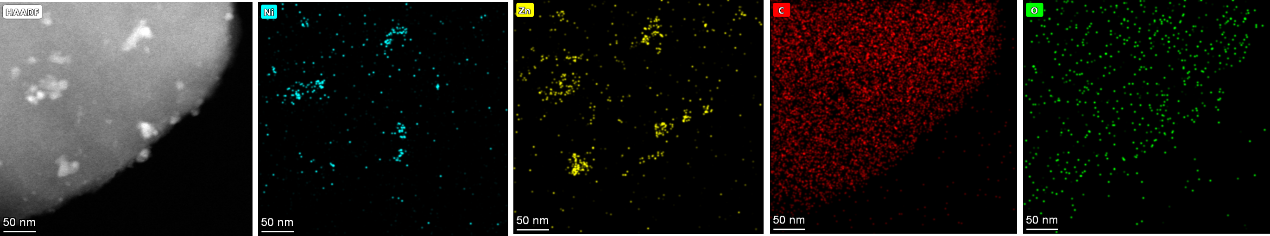


**Fig. S6** HAADF-STEM images of Ni-ZnO/AC


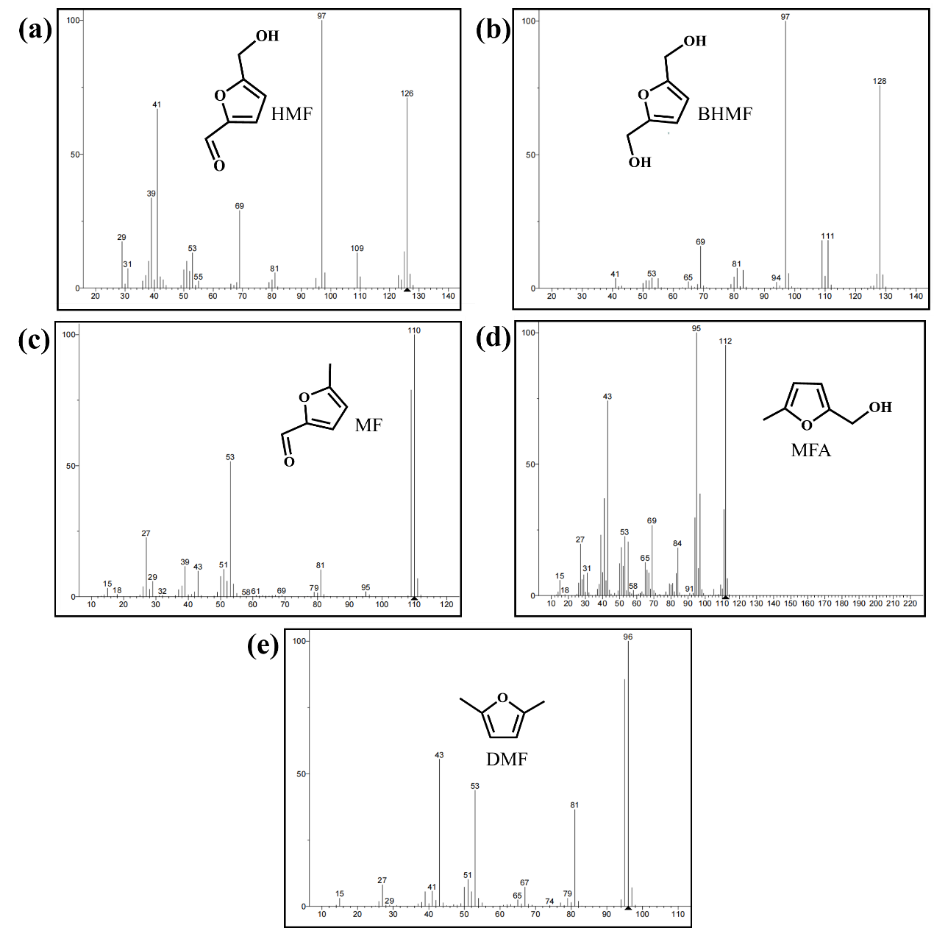


**Fig. S7** GC-MS spectra of the reactant and dominant products in the reaction mixture: **a** HMF, **b** BHMF, **c** MF, **d** MFA and **e** DMF


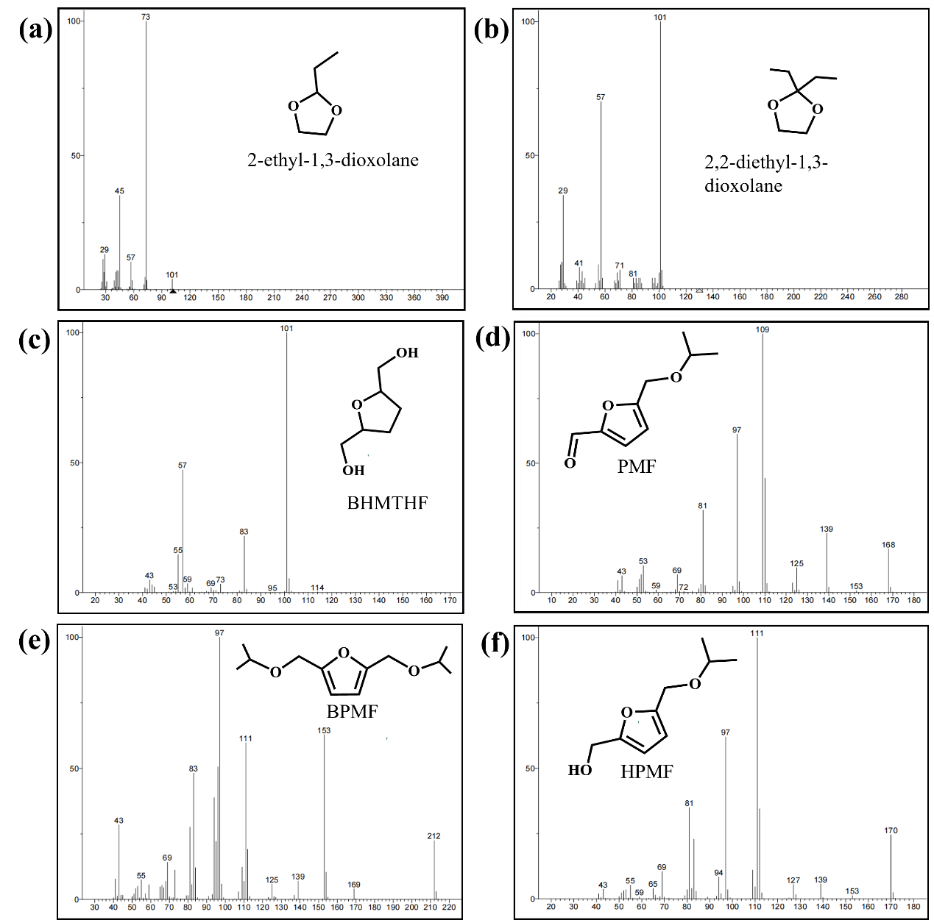


**Fig. S8** GC-MS spectra of by-products in the reaction mixture: **a** 2-ethyl-1,3-dioxolane, **b** 2,2-diethyl-1,3-dioxolane, **c** BHMTHF, **d** PMF, **e** BPMF and **f** and HPMF


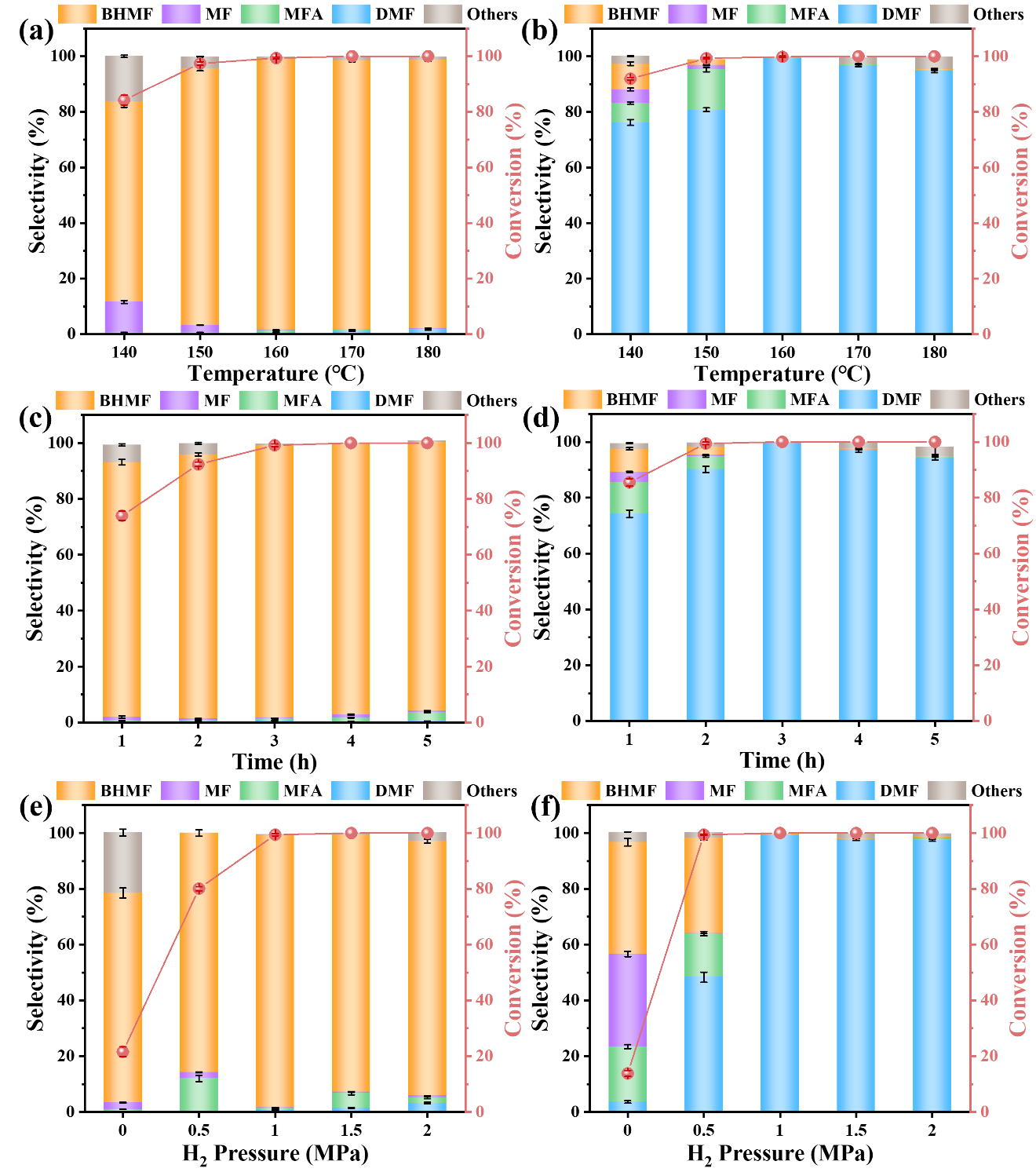


**Fig. S9** Influences of reaction parameters. **a, b** Temperature, **c, d** time, **e, f** H_2_ pressure for selectivity hydrogenation of HMF over Ni-ZnO/AC in 1,4-dioxane (**a, c, e**) and iPrOH (**b, d, f**) solvents, reaction conditions: 0.1 g HMF, 10 mL solvent, 0.05 g catalyst


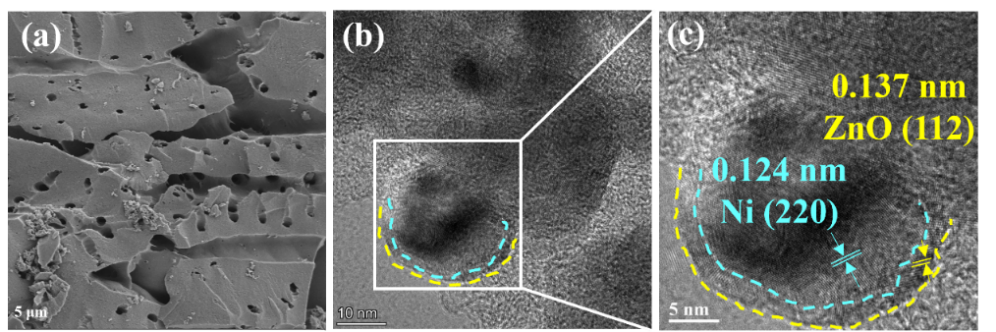


**Fig. S10** **a** SEM and **b, c** TEM images of recovered Ni-ZnO/AC after five cycles


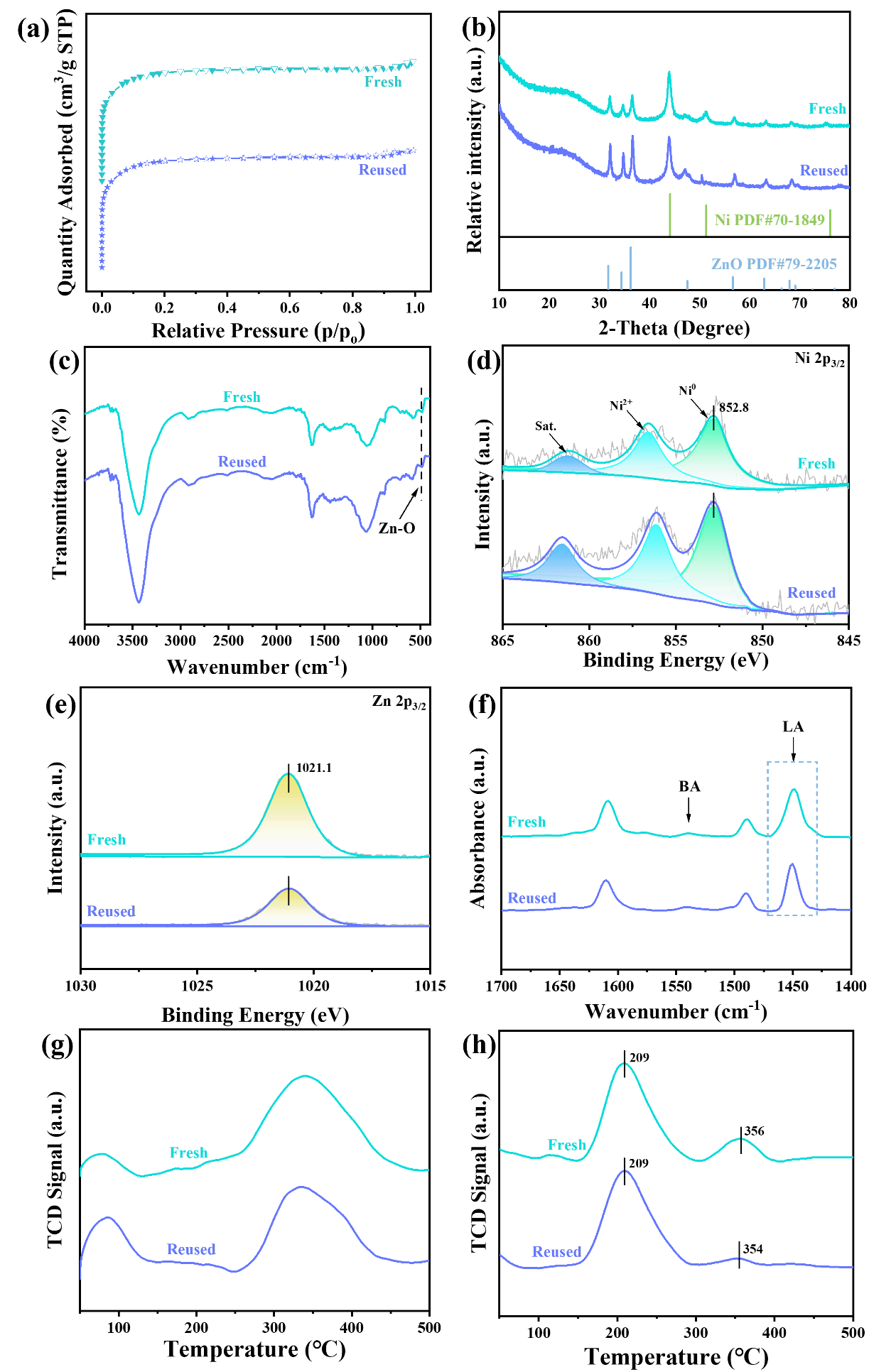


**Fig. S11** **a** N_2_ adsorption/desorption isotherms, **b** XRD patterns, **c** FTIR spectra, **d** Ni 2p_3/2_, **e** Zn 2p_3/2_ XPS spectra, **f** Py-FTIR spectra, **g** NH_3_-TPD profiles and **h** H_2_-TPD profiles of recovered Ni-ZnO/AC after five cycles

**Fig. S12** In-situ FTIR spectrum of BHMF

**
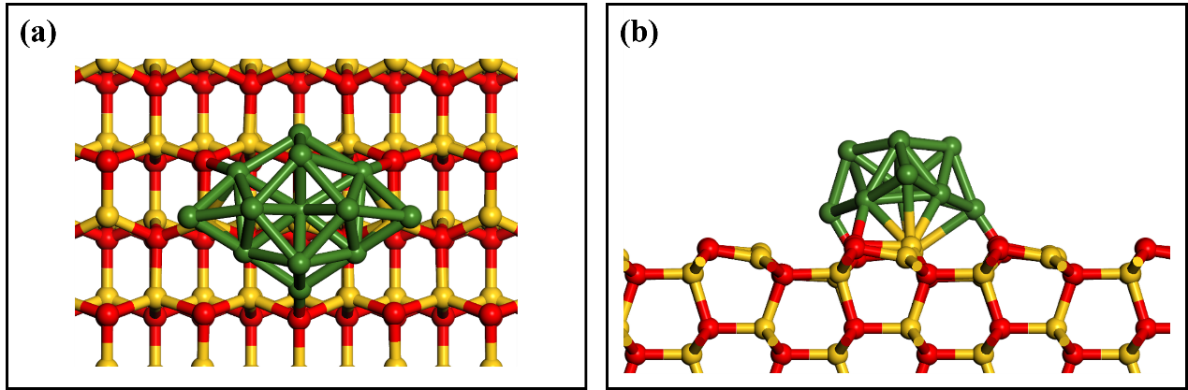
**

**Fig. S13** The as-bulit structural model of Ni-ZnO/AC (**a** for top view and **b** for side view). (Ni: green, Zn: yellow, O: red)


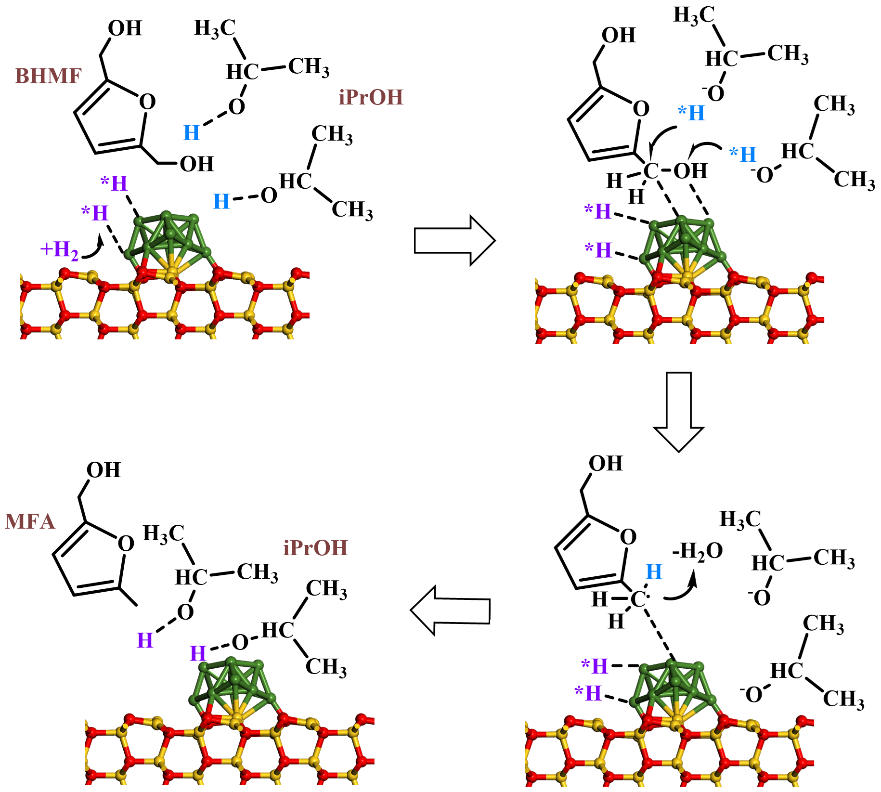


**Fig. S14** Schematic illustration of the hydrogen shuttle mechanism during the HDO step over Ni-ZnO/AC in iPrOH. (Ni: green, Zn: yellow, O: red, to highlight the HDO process, the planar adsorption configuration between the furan ring and Ni sites is simplified)

**Table S1** Physicochemical properties of catalysts

| **Catalyst** | **S_BET_ ^a^**  **(m^2^·g^-1^)** | **V_total_ ^b^**  **(cm^3^·g^-1^)** | **V_micro_ ^c^**  **(cm^3^·g^-1^)** | **Ni content ^d^**  **(wt%)** | **Zn content ^d^**  **(wt%)** |
| --- | --- | --- | --- | --- | --- |
| AC | 1270 | 0.74 | 0.49 | - | - |
| Ni/AC | 1148 | 0.69 | 0.47 | 9.15 | - |
| ZnO/AC | 1100 | 0.64 | 0.42 | - | 13.41 |
| Ni-ZnO/AC | 1243 | 0.67 | 0.44 | 10.09 | 11.02 |
| Ni-ZnO/AC-R5 ^e^ | 1230 | 0.66 | 0.43 | 9.89 | 10.69 |

^a^ Total surface area (S_BET_) determined by BET method.

^b^ Total pore volume (V_total_) obtained according to volume absorbed at P/P_0_ = 0.99.

^c^ Micropore volume (V_micro_) derived from the NLDFT method.

^d^ Ni and Zn content were determined by ICP-OES.

^e^ Recovered catalyst of Ni-ZnO/AC after five cycles.

**Table S2** Composition of acid sites for different catalysts

| **Catalyst** | **Acidity ^a^ (****mmol/g)** |
| --- | --- |
| AC | 0.05 |
| Ni/AC | 0.92 |
| ZnO/AC | 0.54 |
| Ni-ZnO/AC | 1.21 |
| Ni-ZnO/AC-R5 ^b^ | 1.16 |

^a^ Obtained by NH_3_-TPD results.

^b^ Recovered catalyst of Ni-ZnO/AC after five cycles.

**Table S3** Catalytic evaluation of Ni_(x)_-ZnO_(y)_/AC catalysts with different Ni and Zn loadings for selective hydrogenation of HMF in 1,4-dioxane and iPrOH

| **Catalyst ^a^** | **Ni content ^b^**  **(wt%)** | **Zn content ^b^**  **(wt%)** | **Solvent** | **HMF con.**  **(%)** | **BHMF sel.**  **(%)** | **DMF sel.**  **(%)** | **Others sel. ^c^**  **(%)** |
| --- | --- | --- | --- | --- | --- | --- | --- |
| Ni_(5)_-ZnO_(10)_/AC | 4.98 | 10.11 | 1,4-dioxane | 84.2 | 93.8 | 0 | 5.4 |
|  |  |  | iPrOH | 86.1 | 1.2 | 97.7 | 0.9 |
| Ni_(10)_-ZnO_(10)_/AC | 10.09 | 11.02 | 1,4-dioxane | 99.6 | 97.9 | 0 | 0.7 |
|  |  |  | iPrOH | 100 | 0.2 | 99.5 | 0.2 |
| Ni_(15)_-ZnO_(10)_/AC | 14.89 | 9.86 | 1,4-dioxane | 92.3 | 91.1 | 0 | 7.9 |
|  |  |  | iPrOH | 92.8 | 3.6 | 93.4 | 2.5 |
| Ni_(10)_-ZnO_(5)_/AC | 10.04 | 5.03 | 1,4-dioxane | 95.7 | 95.5 | 0 | 3.1 |
|  |  |  | iPrOH | 96.4 | 0.5 | 96.5 | 1.3 |
| Ni_(10)_-ZnO_(15)_/AC | 9.96 | 14.95 | 1,4-dioxane | 82.3 | 90.3 | 0 | 2.6 |
|  |  |  | iPrOH | 84.5 | 5.7 | 92.8 | 0.8 |

^a^ Ni_(x)_-ZnO_(y)_/AC catalysts, x and y represent the theoretical loading amounts of Ni and Zn, respectively. Reaction condition: 0.1 g HMF, 0.05 g catalyst, 1 MPa H_2_, 160 °C, 3 h.

^b^ Ni and Zn content were determined by ICP-OES.

^c^ Other products include MF and MFA.

**Supplementary References**

1. M.J. Frisch, G.W. Trucks, H.B. Schlegel, G.E. Scuseria, M.A. Robb et al., Gaussian 16, Revision C.01, Gaussian, Inc., Wallingford CT, 2016.
2. Roy Dennington, Todd A. Keith, John M. Millam, GaussView, Version 6.1, Semichem Inc., Shawnee Mission, KS, 2016.
3. S. Emamian, T. Lu, H. Kruse, H. Emamian, Exploring nature and predicting strength of hydrogen bonds: a correlation analysis between atoms-in-molecules descriptors, binding energies, and energy components of symmetry-adapted perturbation theory. J. Comput. Chem. **40**(32), 2868–2881 (2019). <https://doi.org/10.1002/jcc.26068>
4. T. Lu, F. Chen, Multiwfn: a multifunctional wavefunction analyzer. J. Comput. Chem. **33**(5), 580–592 (2012). <https://doi.org/10.1002/jcc.22885>
5. W. Humphrey, A. Dalke, K. Schulten, VMD: Visual molecular dynamics. J. Mol. Graph. **14**(1), 33–38 (1996). <https://doi.org/10.1016/0263-7855(96)00018-5>
6. G. Kresse, J. Furthmüller, Efficient iterative schemes for *ab initio* total-energy calculations using a plane-wave basis set. Phys. Rev. B Condens. Matter **54**(16), 11169–11186 (1996). <https://doi.org/10.1103/physrevb.54.11169>
7. G. Kresse, D. Joubert, From ultrasoft pseudopotentials to the projector augmented-wave method. Phys. Rev. B **59**(3), 1758–1775 (1999). <https://doi.org/10.1103/PhysRevB.59.1758>
8. J.P. Perdew, K. Burke, M. Ernzerhof, Generalized gradient approximation made simple. Phys. Rev. Lett. **77**(18), 3865–3868 (1996). <https://doi.org/10.1103/PhysRevLett.77.3865>
9. S. Grimme, S. Ehrlich, L. Goerigk, Effect of the damping function in dispersion corrected density functional theory. J. Comput. Chem. **32**(7), 1456–1465 (2011). <https://doi.org/10.1002/jcc.21759>
10. G. Henkelman, H. Jónsson, Improved tangent estimate in the nudged elastic band method for finding minimum energy paths and saddle points. \jcp **113**(22), 9978–9985 (2000). <https://doi.org/10.1063/1.1323224>
